# The Role of Non-Alcoholic Fatty Liver Disease in Infections

**DOI:** 10.3390/life12122052

**Published:** 2022-12-07

**Authors:** Juraj Krznarić, Adriana Vince

**Affiliations:** 1 Department for Infectious Diseases, School of Medicine, University of Zagreb, 10000 Zagreb, Croatia; 2 Department for Viral Hepatitis, University Hospital for Infectious Diseases, 10000 Zagreb, Croatia

**Keywords:** NAFLD, infectious disease, community acquired pneumonia, COVID-19, *H. pylori*, urinary tract infections, *C. difficile*, bacteremia, hepatitis B, hepatitis C, HIV, periodontitis

## Abstract

Non-alcoholic fatty liver disease (NAFLD) is the most prevalent chronic liver disease, affecting one third of the Western population. The hallmark of the disease is excessive storage of fat in the liver. Most commonly, it is caused by metabolic syndrome (or one of its components). Even though the development of NAFLD has multiple effects on the human organism resulting in systemic chronic low-grade inflammation, this review is focused on NAFLD as a risk factor for the onset, progression, and outcomes of infectious diseases. The correlation between NAFLD and infections is still unclear. Multiple factors (obesity, chronic inflammation, altered immune system function, insulin resistance, altered intestinal microbiota, etc.) have been proposed to play a role in the development and progression of infections in people with NAFLD, although the exact mechanism and the interplay of mentioned factors is still mostly hypothesized. In this article we review only the selection of well-researched topics on NAFLD and infectious diseases (bacterial pneumonia, COVID, *H. pylori*, urinary tract infections, *C. difficile*, bacteremia, hepatitis B, hepatitis C, HIV, and periodontitis).

## 1. Introduction

Non-alcoholic fatty liver disease (NAFLD) is the most prevalent chronic liver disease in the world, linked with multiple systemic changes in immune response, low-grade inflammation, gut dysbiosis and metabolic alterations. Surprisingly, although all these changes play a crucial role in host defense against microorganisms, the role of NALFD in the course and outcome of infections has not been extensively studied. In this review paper, we will focus on the role of NAFLD in infections. The aim of the paper is to review only the well-researched topics on NAFLD and infectious diseases in the hopes of raising awareness of the potential importance of NAFLD as an entity in human pathology.

### 1.1. Defining the NAFLD

Non-alcoholic fatty liver disease (NAFLD) is defined by an abnormal accumulation of fat in the liver with the absence of secondary causes (alcohol use, viral hepatitis, medications). The term NAFLD refers to a range of liver conditions from non-alcoholic fatty liver (NAFL a.k.a. simple steatosis) to non-alcoholic steatohepatitis (NASH) characterized with liver inflammation that can progress to liver cirrhosis and hepatocellular carcinoma [1]. About 30% of patients with NAFLD will develop NASH, and 20% of NASH patients with fibrosis will develop liver cirrhosis [2].

NAFLD is the most common chronic liver disease in the Western population, with the prevalence in imaging studies between 25% and 30%, which is twice as high as that discovered through serum liver marker levels [3]. All continents have high rates of NAFLD; however, the highest rates have been seen in the Middle East (32%) and South America (31%), followed by Asia (27%), USA (24%) and Europe (23%), and less frequently in Africa (14%) [4]. The incidence of NAFLD is lower in younger and older age groups, reaching its peak in adulthood. Due to estrogen deficiency, men and postmenopausal women are at higher risk of developing NAFLD [5,6].

Reduced liver function in NAFLD may be due to a disruption in sinusoid microcirculation, resulting in insufficient hepatic clearance [7]. Furthermore, even in cases of mild steatosis, the immune response of the liver is overly activated, indicating that individuals with NAFLD already have an altered hepatic immune response, resulting in a defective systemic immunological defense against numerous pathogens [8].

Liver biopsy remains the gold standard for identifying and defining the stages of NAFLD. Less invasive methods, such as ultrasonography, fibro elastography and MRI, registers the existence of a fatty liver, but does not measure inflammation or fibrosis reliably [9].

### 1.2. The Pathogenesis of NAFLD and Its Relation to Infectious Diseases

Even though the pathogenesis of NAFLD has not been fully elucidated, the “multiple hit theory” is the most likely explanation. This proposes that several insults, including insulin resistance, high levels of lipopolysaccharides (LPS) in the bloodstream, changes in gut microbiota, genetic and epigenetic factors act together in the genesis of NAFLD [10]. The amount of visceral fat (even in the absence of a high body mass index, BMI and/or waist circumference) seems to be one of the most important risk factors for developing NAFLD. Visceral adipocytes are less developed and more likely to mobilize fat during insulin resistance (IR), which is one of the initial steps in the development of NAFLD. Additionally, visceral fat cells may function as an endocrine organ that secretes cytokines and adipokines that regulate insulin sensitivity and fatty liver accumulation. IR disrupts the metabolism of fat tissue, causing increased lipogenesis in the liver and rapid lipolysis in fat cells resulting in the accumulation of triglycerides in the liver [11,12,13,14].

Higher serum levels of inflammatory biomarkers, such as C-reactive protein (CRP) and interleukins (IL), particularly IL-6, IL-7, leptin, tumor-necrosis factor α (TNF-α), and transforming growth factor β (TGF-β), are found in patients with NAFLD, and their blood levels correlate with NAFLD progression and prognosis [15,16]. In addition, patients with severe liver inflammation have an elevated neutrophil-lymphocyte ratio, which is also considered as a biomarker of NAFLD severity. This leads to uncontrolled cytokine production, elevated IL-6 levels and promotes the differentiation of the Th-17 cell subset [17,18].

Regulatory T cells (Treg) and Th-17 are two CD4+ T lymphocyte subpopulations that develop from T helper lymphocytes. To avoid tissue damage by an uncontrolled immunological response, their interaction helps maintain the proper balance between an immune response to pathogens and immune suppression. Th-17 cells have a potent pro-inflammatory role; they release IL-17, IL-21, and IL-22, which are involved in a variety of chronic diseases, as well as the host’s immunological response to infection. A pro-inflammatory IL-17 drives liver fibrosis, neutrophil activation, and the release of numerous other inflammatory mediators such as TNF-α, IL-1, and IL-6. The increased generation of pro-inflammatory cytokines promotes Th-17 differentiation, leading to a hyperactivation of the Th-17/IL-17 system, which further drives liver inflammation and fibrinogenesis [17]. Furthermore, patients with NAFLD have lower levels of Th-17 inhibitory cytokines (IL-10, IL-4, IL-22, and interferon γ, IFN-γ). Notably, one of the key elements contributing to the development of multiorgan dysfunction and sepsis outcomes is the imbalance between Treg and Th-17 [18,19].

Furthermore, the innate immune system protein complement component 3 (C3), which plays a crucial role in the function of the immune system, may be positively correlated with the severity of NAFLD. Bacterial endotoxins, free fatty acids (FFA), and cholesterol can trigger the complement system in the early stages of NAFLD through danger-associated molecular patterns (DAMPs) or pathogen-associated molecular patterns (PAMPs). Several studies showed that serum concentrations of C3 in NAFLD patients are significantly higher than in healthy controls, and their concentrations correlate with disease severity. The underlying mechanism for complement C3 in NAFLD is still not completely understood. Some studies suggest that C3 regulates lipid metabolism and excessive fat storage. The C3a/C3aR signaling axis often plays a pro-inflammatory function. In NASH, C3a promotes inflammation by activating Kupffer cells in response to neutrophil elastase [19]. The development of organ ischemia-reperfusion injury, sepsis, and metabolic inflammation is significantly influenced by C3a [20,21].

In NAFLD, overactive Kupffer cells secrete multiple inflammatory cytokines (including TNF-α, IL-1, and IL-6), as well as inflammatory chemokines (such as CCL2, CCL3, CCL5, CXCL16, CX3CL1). Kupffer cells additionally attract a significant number of bone marrow-derived monocytes/macrophages into the liver causing subsequent hepatic inflammation, finally leading to NASH [22]. For a simplified overview of the role of acute phase proteins in NAFLD, please see Table 1.

**Figure 1:**
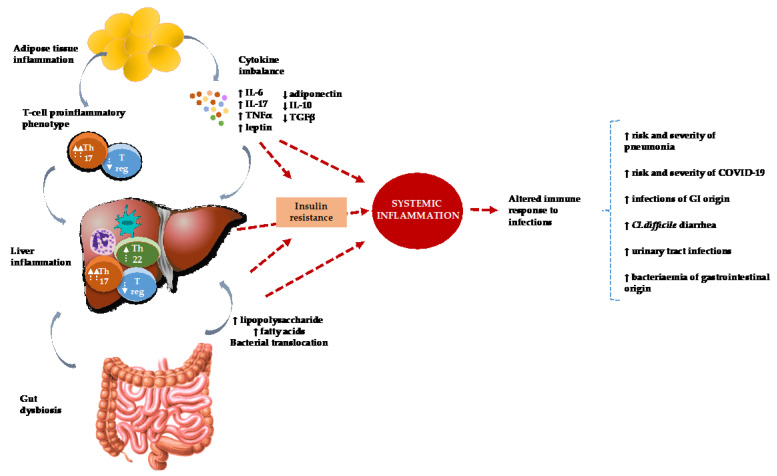
Immunological pathways that associate NAFLD and infections.

Since the liver is responsible for a vast array of physiological processes, such as detoxification, energy production, nutrition processing, hormonal balancing, immunological response, and coagulation, it is also a crucial organ for surviving infections. In addition, it plays a pivotal role in the removal of bacteria and toxins from the bloodstream. In bacterial infections, lipopolysaccharides (LPS), which are metabolized in the liver, are one of the major activators of the inflammatory response [29,30]. Additionally, the liver produces acute phase proteins of the inflammatory response that contribute to the systemic activation of the immune response by functioning as opsonins, activating neutrophils and/or macrophages, and regulating the antimicrobial capabilities of the complement system [16]. Moreover, the liver produces anti-inflammatory mediators including interleukin 10 (IL-10), transforming growth factor (TGF-β), and glucocorticoids. An equilibrium between the inflammatory and anti-inflammatory responses leads to the removal of bacteria and resolution of inflammation, resulting in the recovery from sepsis. It is also worth mentioning that the mortality rate in sepsis complicated by acute renal or respiratory insufficiency is lower than in sepsis accompanied by liver failure [19,31,32]. See Figure 1 for a graphic view of immunological pathways and their associations with NAFLD and infections.

Another possible factor in the development of NAFLD is vitamin D deficiency. Vitamin D’s “pleiotropic” effects, which include roles in immunological modulation, cell differentiation and proliferation, and regulation of inflammation, have been connected to the etiology and severity of NAFLD. While some studies have found a link between vitamin D and NAFLD/NASH, other studies have not been able to. Therefore, the direct association of vitamin D deficiency and NAFLD is still uncertain. The role of vitamin D in NAFLD/NASH would be made clearer by future large, randomized controlled studies of vitamin D treatment alone in populations with various clinical and metabolic risk factors for vitamin D deficiency and NAFLD, as well as various subtypes of steatosis, NASH, and liver fibrosis [33].

## 2. Association between NAFLD and Infectious Diseases

### 2.1. NAFLD and Community Acquired Pneumonia

Community-acquired pneumonia (CAP) is a major cause of morbidity and mortality worldwide [34]. There have only been a few studies evaluating the relationship between NAFLD and CAP.

Nseir et al. published a case-control retrospective study in 2017 that included 141 patients who were hospitalized for the treatment of CAP during a 3-year period. The patients with CAP were older than 18 years and had imaging data of the liver by abdominal ultrasonography. Structural lung disease and immunocompromised patients were excluded. A control group was made up of 141 patients who were hospitalized in the same study period matched for age, gender and BMI, and no evidence of current infectious disease. A total of 40.4% of the subjects from the study group showed evidence of NAFLD vs. only 27.6% from the control group. Mean CRP levels were significantly higher in patients with CAP. A multivariate analysis showed that NAFLD was associated with CAP. This was the first study to find this association [35]. Unfortunately, the study did not stratify the severity of liver disease and its correlation with the severity of CAP and its outcome.

The same group later reported a retrospective cohort study including 561 patients with CAP and assessed the impact of NAFLD on CAP survival. The prevalence of NAFLD was 35.6%. Significant differences were found between the NAFLD and non-NAFLD group in BMI, CURB-65 (a clinical prediction rule for the purpose of predicting mortality in community-acquired pneumonia including five risk factors, each worth one point: confusion of new onset, blood urea nitrogen greater than 7 mmol/L (19 mg/dL), respiration rate of 30 breaths per minute or above, blood pressure less than 90 mmHg, systolic or diastolic blood pressure of 60 mmHg or less and age of 65 or older), ALT, GGT, and CRP. The mortality in the NAFLD group was 17%, and in controls it was 5.8%. In multivariate logistic regression analysis, NAFLD with fibrosis score 0–2, NAFLD with fibrosis score > 2 were associated with 30-day all-cause mortality, independently of other components of metabolic syndrome [36].

Several other studies examined the impact of T2DM or obesity on CAP outcomes, however none of them included NAFLD as a variable. Patients with T2DM have reduced neutrophil function (thereby increasing their likelihood of acquiring infectious illnesses) and a generally poorer prognosis for CAP (increased rating of pleural effusions and mortality) [37,38]. There appears to be no substantial difference in mortality between obese patients with pneumonia and normal-weight patients, even though obese patients are more susceptible to respiratory tract infections. However, studies examining the relationship between obesity, T2DM, and pneumonia demonstrates that obese people with T2DM have a higher incidence and severity of infection [39,40].

The term “obesity paradox” (a lower mortality rate for overweight or obese people within certain subpopulations) has also been recorded by some researchers in patients with CAP [41]. This finding is mainly based on observational studies that might be biased by patients’ selection since obese patients more frequently develop CAP and they might be overrepresented in cohorts [41]. Another possible explanation includes so called reversal causation, where normal weight patients might have other risk factors for worse outcomes [41]. One of the interesting research topics is the alteration of gut microbiota in obese patients that might modulate immune response and have an impact on survival. This connection depends on the gut metagenome, and changes in the microbial population might result in modifications to the normal metabolism. Additionally, it has been discovered that the gut microbiota influences lipid metabolism, inflammation, and atherogenesis via lipopolysaccharides and peptidoglycans [42]. Further research on this topic is necessary.

### 2.2. NAFLD and COVID-19

There is growing evidence that NAFLD is a risk factor for acquiring the SARS-CoV2 infection. Angiotensin-converting enzyme 2 (ACE2), a cellular entrance receptor for SARS-CoV-2, is also present in the hepatobiliary and gastrointestinal epithelial cells, making the entire gastrointestinal (GI) system susceptible to infection [43].

In patients with NASH, the expression of genes that enhance the affinity of coronaviruses for hepatic tissue is also elevated. This also suggests that people with advanced NAFLD may be even more susceptible to COVID-19 [44]. Additionally, compared to patients without NAFLD, those with NAFLD seem to have a greater chance of disease progression and a longer viral shedding time [45].

Younossi et al. used electronic medical record data of adult COVID-19 patients hospitalized between March and December 2020 to identify the determinants of mortality and hospital resource consumption among patients with NAFLD. Imaging methods or a liver biopsy were used to detect NAFLD in the absence of other liver disorders. Out of the 4835 patients hospitalized with COVID-19, 553 had NAFLD. Of those, 58% were obese, 15% were morbidly obese (BMI > 40kg/m^2^), 51% had T2DM, and 63% had arterial hypertension. When compared to patients without NAFLD, patients with NAFLD experienced more pronounced respiratory symptoms, a higher body temperature and heart rate, and higher levels of alanine and aspartate aminotransferases. Only 3.9% of individuals with NAFLD had acute liver damage. With a crude inpatient mortality rate of 11%, the NAFLD group had considerably longer lengths of stay, more ICU admission, and they more frequently required mechanical ventilation. Older age, morbid obesity, a higher Fibrosis-4 Index (FIB-4) score were independent predictors of mortality in patients with NAFLD, but not sex, race/ethnicity, or other comorbidities. The study’s authors concluded that patients with NAFLD present with a more severe disease at admission and demand more hospital resources [46].

Between February and April 2020, all consecutive patients hospitalized with COVID-19 were enrolled in a cohort study undertaken by Forlano et al. Patients were categorized based on their FIB-4 index and imaging results. The study involved 193 patients. A total of 59 patients (30%) died, nine (5%) remained in the hospital, and 125 (65%) were released. When compared to the non-NAFLD cohort (*n* = 132), the NAFLD cohort (*n* = 61) was younger (60 vs. 70.5 years). The diagnosis of NAFLD was not linked to worse outcomes. However, the NAFLD group had higher CRP levels. While intermediate/high risk FIB-4 or liver cirrhosis were not linked with in-hospital mortality among NAFLD patients, male gender, ferritin, and early warning score (EWS) were. Mortality in the NAFLD group was associated with male gender and inflammatory response [47].

Early in 2020, Ji et al. reported data on 202 consecutive patients with NAFLD (based on HSI index) and COVID-19 from two China-based hospitals. On admission and throughout hospitalization, liver damage was detected in 101 (50%) and 152 (75.2%) individuals, respectively. Only 2.6% (4/152) of liver injuries exhibited ductular or mixed patterns, making up most hepatocellular injuries. From admission to discharge, 67 patients (33.2%) had persistently abnormal liver function. Of total patients, 163 (80.7%) and 39 (19.3%) had stable disease, whereas 39 had progressing disease. Patients with disease progression tended to be older, had higher BMIs, higher comorbidity rates, and NAFLD. When compared to patients without NAFLD, patients with NAFLD had a higher likelihood of impaired liver function from admission to discharge, and a longer viral shedding period. One of the patients’ postmortem liver biopsies revealed only microvesicular steatosis and overactive T cells, indicating that the liver damage in COVID-19 is probably immune-mediated [48].

Electronic medical record data of 6700 persons with a positive SARS-CoV-2 PCR between 1 March 2020, and 25 August 2020 were retrospectively analyzed by Bramante et al. The probabilities of hospital admission were calculated using logistic regression and competing risk. A history of NAFLD/NASH was associated with increased odds of admission for COVID-19. After adjusting for NAFLD/NASH, people who were obese had lower risks of being hospitalized for COVID-19. In all racial/ethnic categories, including men and women, NAFLD/NASH increased hospitalization risk [49].

Adult patients with severe COVID-19 who were consecutively hospitalized between March and June 2021 were included in the prospective observational study by Vrsaljko et al. A total of 120 of the 216 included patients had NAFLD. The C-reactive protein, interleukin-6, aspartate aminotransferase, alanine aminotransferase, and lactate dehydrogenase levels were higher in the NAFLD group. Patients with NAFLD more frequently required noninvasive ventilation or high-flow nasal cannulas, had longer hospitalization, and were more frequently diagnosed with pulmonary thromboembolism. NAFLD was found to be a risk factor for pulmonary thrombosis and to have a negative correlation with time to recovery [50]. This study also did not stratify the severity of liver disease and its correlation with the severity of COVID and its outcome.

The two primary ways by which SARS-CoV-2 affects the liver are direct cytopathic damage induced by the virus itself, and indirect inflammation resulting in hepatic ischemia or the exacerbation of preexisting liver illness. Typically, both mechanisms are present at the same time [50]. Another mechanism of liver damage is drug-induced liver injury (DILI) due to the treatment, which is also relatively common in patients with preexisting liver disease [51]. COVID-19 is characterized by a hepatocellular pattern of liver abnormalities. Associations of NAFLD with COVID-19 outcomes might be the result of persistent lipotoxicity, chronic inflammation, insulin resistance, oxidative stress, and immunological response. Additionally, IL-6 (which is typically elevated in people with NAFLD) is overproduced during COVID-19 infection, resulting in a ‘cytokine storm’ that exacerbates the organism’s inflammation [44,45,46,47,48,49,50,51,52].

### 2.3. NAFLD and H. pylori

The human stomach is commonly colonized by the Gram-negative, microaerophilic bacterium *Helicobacter pylori*. The colonization is approximately 20% prevalent in wealthy nations and up to 70% prevalent in developing nations. While some research implies a possible link between *H. pylori* and NAFLD, others do not support it. Also, the underlying pathogenic process is not entirely understood. This suggests that the eradication of *H. pylori* may play an important role in the treatment of NAFLD [16].

According to a recent meta-analysis, *H. pylori* infection was associated with an increased incidence of NAFLD. *H. pylori* was an independent risk factor for NAFLD related to a higher degree of steatosis. The *H. pylori*-positive group, however, had a considerably higher proportion of patients with arterial hypertension, as well as higher BMI, total cholesterol, triglycerides, and LDL levels, while having lower HDL values.In addition, a recent study found that individuals with NASH who test positive for *H. pylori* are more likely to have hepatocyte ballooning. This shows that *H. pylori* may not be directly related with NAFLD but may contribute to the progression of the disease to NASH [53,54].

### 2.4. NAFLD and Urinary Tract Infections

The studies examining the association between NAFLD and urinary tract infections (UTIs) are limited.

Nseir et al. conducted a study in 2019 on the association between NAFLD and recurrent urinary tract infections (rUTI) in premenopausal women without metabolic syndrome. This was a 3-year retrospective case-control study including 1009 premenopausal women hospitalized and treated for UTI. Ultimately, 372 participants were enrolled in the trial (186 participants with rUTI and 186 controls without a history of rUTI). As part of the inclusion criteria, abdominal ultrasonography was performed on each subject. The two groups were compared to identify factors associated with rUTI, including maternal history of rUTI, use of contraceptives, frequency of sexual activity, metabolic syndrome, obesity, usage of probiotics, serum vitamin D levels, and NAFLD. A recurrent UTI was defined as three or more bouts of UTI within one year. Mean age of the 372 participants was 39.7 ± 5 years. NAFLD was diagnosed in 43.5% of subjects with rUTI vs. 21.5% controls. Women with rUTI were more often obese and presented with lower serum levels of vitamin D. A multivariate analysis showed that NAFLD was associated with rUTI in premenopausal women independent of metabolic syndrome [55]. This study also did not stratify the severity of liver disease and its correlation with the severity of UTIs and their outcomes.

A few studies have also examined the association between NAFLD and urolithiasis. All have established that NAFLD is an independent risk factor for the development of urolithiasis. Given that urolithiasis is a predisposing factor for UTI, this is an important finding [56].

Studies that investigated the association between serum 25(OH)D concentrations and NAFLD found that persons with NAFLD have lower serum 25(OH)D concentrations. This is intriguing because reduced blood 25(OH)D levels have a direct effect on the occurrence of a higher rate of rUTI [57,58].

### 2.5. NAFLD and C. difficile

Due to the high correlation between *C. difficile* associated diarrhea (CDAD) and prior antibiotic usage, it has been determined that disruptions in the gut flora are a key factor in CDAD. Proton pump inhibitor use and advanced age are also risk factors for acquiring CDAD. These elements are linked to modifications in the gut microbiota’s constitution. Studies have revealed a link between lower microbiota diversity and the presence of *C. difficile*, either as a colonizer or as a pathogen. Changes in the representation of microbial populations (e.g., taxa) appear to be linked to *C. difficile* infection per se and may either function to increase susceptibility to *C. difficile* infection or to act as a barrier to *C. difficile* colonization of the gut [59].

NAFLD was reported as an independent predictor for the development of *C. difficile* associated diarrhea (CDAD).

Papić et al. conducted a study in 2019 which identified NAFLD as an independent predictor of CDAD. This was a retrospective cohort study that included patients ≥ 65 years, treated with antimicrobial therapy ≥ 24 h, and hospitalized ≥ 72 h during a 36-month period. Of the 314 patients included in the study, 83 had NAFLD. Diabetes and obesity were more common in the NAFLD group. A total of 16% of the patients with NAFLD and 7.4% of patients in the control group developed in-hospital CDAD. The study concluded that NAFLD is an independent predictor of CDAD [60].

Nseir et al. conducted a similar study in 2020. This was a retrospective study of patients admitted to the hospital for CDAD during a period of 4 years. The control group consisted of patients with CD toxin (CDT) negative diarrhea. The controls were matched for age and gender. A total of 230 patients were included in the study (115 CDT positive, 115 CDT negative). The mean age was 69.57 ± 18 years. NAFLD was found in 66% of patients with CDAD vs. 30.4% with CDT negative diarrhea. In addition, the CDAD group had significant associations with metabolic syndrome. A multivariate analysis showed that NAFLD is significantly associated with CDAD [61].

In 2021 Šamadan et al. published a study on NAFLD being a risk factor for recurrent CDAD. This was a retrospective cohort study that included patients ≥ 60 years hospitalized with CDAD. The cohort was divided into two groups: those who were and were not readmitted with CDAD within 3 months of index discharge. Of the 329 patients included, 32.5% experienced recurrent CDAD. Chronic kidney disease and NAFLD were also more common in this group, with no other major differences in the two groups. Analysis showed that age > 75 years, NAFLD, Charlson Age–Comorbidity Index (CACI) > 6, chronic kidney disease, statins and immobility were associated with recurrent CDAD, making NAFLD a possible host-related risk factor associated with recurrent CDAD [62]. These studies did not stratify the severity of liver disease and its correlation with the severity of CDAD and its outcome.

### 2.6. NAFLD, Bacteremia and Recurring Bacterial Infections

One of the first studies to assess this was conducted by Nseir et al. (2011). A total of 296 hospitalized NAFLD patients were assessed over a three-year period for the occurrence of recurrent bacterial infections (RBI) and were compared with 100 age and gender-matched patients without NAFLD who were hospitalized over the same period due to non-recurrent bacterial infections. NAFLD patients had significantly more RBIs than the patients without NAFLD (22% vs. 8%). Analysis showed that age, BMI, male waist circumference, serum 25(OH)D, triglycerides, serum malondialdehyde and paroxonase-1 are associated with RBIs in NAFLD patients. It is important to mention that these factors were associated with RBIs, irrespective of metabolic syndrome [63].

NAFLD patients have increased intestinal mucosal permeability in comparison to healthy people [64]. Small intestine bacterial overgrowth contributes to the etiology of mucosal permeability, as exogenous and endogenous causes can change the gut microbiome. Modulation of the gut microbiota is associated with increased intestinal permeability, which precedes the onset of metabolic endotoxemia, inflammation, and associated diseases. Gut dysbiosis may result in the translocation of gut bacteria into the circulation [65].

Nseir et al. did a study in 2016 on the relationship between primary bacteremia (PB) with a likely GI origin and NAFLD. At least two positive cultures of *Salmonella enterica*, *Proteus*, *Klebsiella* spp., *Citrobacter*, *Escherichia coli*, and *Enterococci* were found in PB that was thought to have originated from the gastrointestinal system. The presence of fatty liver by ultrasonography and the lack of secondary causes of NAFLD were used to make the diagnosis of NAFLD. In total, 946 distinct cases of bacteremia were examined and 14% had PB. Out of them, 71 patients with PB and hepatic ultrasonography were included. Between the two groups with and without NAFLD, there were no differences in the mean age, CRP, DM, chronic renal failure, or malignancy. However, there was a substantial difference in the proportion of women, obesity, and PB thought to originate from the GI tract. Most patients with PB were found to have NAFLD (68.5%). The most common bacteria in the NAFLD group (77%) of patients was *Escherichia coli* (50% of patients in the group without NAFLD had *E. coli*). This retrospective analysis of PB cases revealed a correlation between NAFLD and PB in patients with PB thought to have gastrointestinal origin [66].

Gjurašin et al. examined mortality in patients with NAFLD and invasive group B streptococcus (GBS) disease. This study analyzed non-pregnant adults who were diagnosed with GBS infection over a 15-year period. This was a retrospective cohort analysis. Cellulitis/erysipelas (34.3%), pneumonia (12.7%), endocarditis (7.8%), and bacteremia without a specified source (36.3%) were the most common syndromes among the 102 patients who entered the study. Diabetes (41.2%), dyslipidemia (38.2%), cardiovascular disease (33.3%), peripheral vascular disease (20.6%), obesity (20.6%), and cancer (9.8%) were the most prevalent comorbidities. The patients were divided into two groups based on the findings of the abdominal ultrasound: those with steatosis (43.1%) and those without steatosis (56.9%). Clinical presentations and comorbidities between groups did not differ significantly. Patients with NAFLD had in-hospital mortality rate of 29.5%, compared to 10.3% in the control group. Acute renal failure, qSOFA ≥ 2, endocarditis, and NAFLD were all independently linked to in-hospital mortality. The study concluded that NAFLD is associated with higher mortality in individuals with invasive GBS disease [67]. These studies did not stratify the severity of liver disease and its correlation with the severity of bacteremia, RBIs and their outcome.

### 2.7. NAFLD and Hepatitis B, C and HIV

Liver steatosis occurs in approximately 50% of patients with HCV, while NASH is found in 10%. Because HCV genotype 3 directly induces fatty liver deposition, it is associated with the highest prevalence and severity, whereas other HCV genotypes demonstrated a lower prevalence of steatosis. In general, HCV alters lipid and glucose metabolism, leading to the accumulation of fats in the liver. It has also been pointed out by Adinolfi et al. that HCV-associated NAFLD has accelerated development of NASH, hepatic fibrosis, and ultimately hepatocellular carcinoma (HCC). This is due to elevated liver inflammation and oxidative stress levels. This environment also leads to HCV persistence and replication. Some extrahepatic symptoms of chronic HCV infection are influenced by HCV-associated steatosis (diabetes, metabolic syndrome, and atherosclerosis) [68].

Similarly, human immunodeficiency virus (HIV) infection also results in fatty liver due to various viral and host factors, as well as the anti-viral medications used to treat HIV. Liver disease is one of the primary causes of death among HIV-positive individuals, particularly if they are also coinfected with HCV. Development of insulin resistance, the release of free fatty acids from adipose tissue, hepatic triglyceride deposition, and oxidative stress-cytokine-mediated damage explains the mechanism. The high incidence of lipid and glucose abnormalities is a consequence of HIV infection and/or antiretroviral medications (some of which cause direct hepatotoxicity and steatosis). HIV also causes persistent inflammation in the majority of infected individuals. HIV-infected individuals have a prevalence rate of approximately 30% for NAFLD, whereas those coinfected with HCV have an incidence rate of 40–72%. In their study, Maurice et al. concluded that, possibly independent of dysbiosis and intestinal translocation, monocyte activation associated with central adiposity appears to be a critical factor in the development of NAFLD and severe liver fibrosis in HIV-monoinfected patients [69].

On the other hand, Joo et al. have demonstrated that HBsAg positivity is associated with a reduced chance of developing NAFLD, indicating an inverse connection between hepatitis B (HBV) infection and NAFLD. In contrast to HBsAg-negative participants, HBsAg-positive subjects experienced a considerable decline in their total cholesterol levels over time. The effect of HBV on steatosis is still not well understood [70].

### 2.8. NAFLD and Periodontitis

Gram-negative bacteria present in dental plaque are the main cause of most periodontal diseases. It is generally recognized that *Porphyromonas gingivalis* (*P. gingivalis*) causes periodontitis [71].

Yoneda et al. showed that NAFLD patients had considerably higher detection rates of *P. gingivalis* (46.7% vs. 21.7%) than non-NAFLD controls. Additionally, *P. gingivalis* was found more frequently (52%) in NASH patients than in participants without NAFLD. The majority of *P. gingivalis* fimbria found in NAFLD patients belonged to the invasive genotypes, particularly type II (50.0%). The three-month non-surgical periodontal therapies for NAFLD patients improved the liver function measures, including the serum AST and ALT levels [60]. This finding implies that *P. gingivalis* infection may be implicated in the development of NAFLD because *P. gingivalis* or the endotoxins produced by the bacteria can easily reach the bloodstream. In addition, *A. actinomycetemcomitans* is frequently discovered in severe periodontitis. According to research, the administration of *A. actinomycetemcomitans* to test animals has been linked with an increase in metabolic diseases [72].

## 3. Conclusions

The liver is essential for host defense against infectious diseases. Multiple studies have demonstrated that the more severe the liver dysfunction, the weaker the host’s immune response and, consequently, the survival fitness of the host (see Table 2). Since NAFLD is considered an early stage of liver disease and is a relatively newly recognized medical syndrome, there is limited research examining its impact on the onset, progression, and prognosis of infectious diseases. Existing evidence indicates that patients with NAFLD have a higher rate of infections, a longer and more complicated infection course, and a higher fatality rate compared to those with a healthy liver. The reason appears to be the chronic low-grade inflammation that can gradually damage the liver’s architecture and, consequently, its immunologic function. Also, NAFLD negatively modifies the gut microbiota, resulting in a variety of gut-related bacterial complications. It should be noted that the low-grade inflammation does not appear to be restricted to the liver, but rather widespread throughout the body, causing alterations in nearly all organ systems. However, studies on the topic are limited, mainly retrospective, and only include a small number of patients. In addition, most of the patients observed in these studies did not only have NAFLD, but they also had one or more components of metabolic syndrome (most commonly DM) and varying stages of liver disease (from different grades of NAFLD to liver cirrhosis), which was not always specified or considered in the mentioned studies. Therefore, it is important to further explore the association between patients with only NAFLD (its stage of severity) and infectious diseases in larger multi-center prospective studies, as a greater understanding of the problem could lead to the development of treatment and preventative measures.

## Figures and Tables

**Table 1:** The role of acute phase proteins in NAFLD.

Acute Phase Proteins	Biological Modifiers	Immune Function	Role in NAFLD
Inflammatory proteins	IL-6, lipopolysaccharide-binding protein, and secreted phospholipase A2, C-reactivee protein, manose binding lectin, collectin liver 1, ficolin-2, serum amyloid P, serum amyloid A	Enhance pro-inflammatory signals and potentiate acute response; secreted pathogen recognition receptors; activation of complement enhanced phagocytosis	Increased levels of CRP, IL1β, IL-6, TNF-α, and ICAM-1 are strongly linked to an increased risk of developing NAFLD [19].
Complement proteins	C3, C4, C9, C4b-binding protein, mannose-binding lectin, C1 esterase inhibitor	Enhanced phagocytosis, chemo attractants, neutrophil degranulation; bacterial cell wall lysis; vascular permeability	The production of C3a and C5a because of complement system activation has been linked to both insulin resistance and pro-inflammatory activity. By controlling the hepatic inflammatory response, the complement system also contributes to NASH [20].
Negative acute phase proteins	Albumin, transferrin, transthyretin, retinol-binding protein, antithrombin, transcortin, insulin like growth factor 1, A2 HS glycoprotein, alpha-fetoprotein, factor XII	Homeostasis, metabolism, transporter proteins	A reduction in serum albumin correlates with a NAFLD severity [23]. As the degree of hepatic steatosis increases, AFP levels rise [24].
Protease inhibitors	α 2 macroglobulin, α 1 antitrypsin, α 1 antichymotrypsin	Anti-inflammatory role	Hyperferritinemia and sinusoidal iron accumulation are linked to α 1 antitrypsin mutations in NAFLD [25].
Antimicrobial proteins	Liver expressed antimicrobial peptide 2, hepcidin	Antimicrobial activity	LEAP2 is linked to steatosis and the lipolytic/lipogenic pathway [26].
Clotting factors	Fibrinogen, plasminogen, protein S, prothrombin, factor VIII, factor IX and Von Willenbrand factor, vitronectin	Coagulation and fibrinolysis	Some changes in hemostatic parameters are anticipated in all forms of liver disease, including NAFLD [27].
Iron-binding proteins	Haptoglobin, hemopexin, ferritin, hepcidin	Reduction of free iron in the serum; antimicrobial functions	The amount of body iron corresponds with serum hepcidin in the liver but not with the severity of steatohepatitis or lipid status [28].

**Table 2:** A simplified view of the current research pertaining to NAFLD and its relationship to infections.

	Study Design	Study Methodology	Main Findings	Comment
**Bacterial pneumonia**				
Nseir et al. [35]	case-control retrospective study	141 patients hospitalized for the treatment of CAP	- 40.4%showed evidence of NAFLD - mean CRP levels significantly higher in patients with CAP - NAFLD was associated with CAP	- mechanisms responsible for increased incidence of bacterial infections in patients with fatty liver unclear - possible alterations in the immune system
Nseir et al. [36]	retrospective cohort study	561 patients with CAP	- the prevalence of NAFLD was 35.6% - the mortality of the NAFLD group was 17% and in controls 5.8%	- NAFLD with fibrosis score > 2 were associated with 30-day all-cause mortality
**COVID-19**				
Younossi et al. [46]	retrospective	553 patients hospitalized with COVID-19 and NAFLD and a baseline Elixhauser comorbidity score of 13.6	- patients with NAFLD experienced greater respiratory symptoms, higher body temperature, heart rate, higher levels of ALT and AST - 3.9% had acute liver damage - longer lengths of stay - more usage of ICUs and mechanical breathing	- COVID-19-infected patients with NAFLD tend to be sicker upon admission and demand more hospital resources-higher FIB-4 and multimorbidity scores, morbid obesity, older age, and hypoxemia at admission were all independent predictors of mortality
Forlano et al. [47]	cohort study	193 patients with COVID	- presence of NAFLD was not associated with worse outcomes in patients hospitalized for COVID-19	- NAFLD patients were younger on admission - disease stage was not associated with clinical outcomes - mortality was associated with gender and a pronounced inflammatory response in the NAFLD group
Ji et al. [48]	cohort study	202 consecutive patients with confirmed COVID-19 and information relating NAFLD status	- male sex, age > 60 years, higher BMI, underlying comorbidity and NAFLD were associated with COVID-19 progression	- pattern of liver injury was mostly hepatocellular - biliary cells have high expression of ACE2 receptor with a high affinity to the spike protein of SARS-CoV-2
Bramante et al. [49]	retrospective analysis	6700 adults with a positive SARS-CoV-2 PCR had assessed odds of hospital admission	- NAFLD/NASH is a significant risk factor for hospitalization for COVID-19 - also appears to account for risk attributed to obesity	- treatments for metabolic disease mitigated risks from NAFLD/NASH
Vrsaljko et al. [50]	prospective observational study	216 adult patients hospitalized with severe COVID were also assessed for NAFLD	- NAFLD is linked to worse COVID-19 outcomes, more pulmonary thromboses, and worse COVID-19 severity	patients with NAFLD typically required noninvasive ventilation or high-flow nasal cannulas more frequently, spent longer time in hospitals
***H. pylori***				
Baeg et al. [53]	retrospective	3663 people were analyzed, 1636 (44.7%) were *H. pylori* positive	- the percentage of people with NAFLD did not differ between infected and uninfected groups	- *H. pylori* infection is not a risk factor for NAFLD
Abo-Amer et al. [54]	cross-sectional study	Of 646 patients; *H. pylori* infection was found to be present in 538 patients (83.3%).	- NAFLD, ALT, AST, hepatomegaly, hypertension, fasting blood sugar significantly higher in *H. pylori* positive group than *H. pylori* negative group	- *H. pylori* infection was independent risk factor for NAFLD and correlated with increased degree of steatosis
**UTI**				
Nseir et al. [55]	retrospective case-control study	186 participants with rUTI and 186 controls without a history of rUTI	- NAFLD was diagnosed in 43.5% of subjects with rUTI	- NAFLD was associated with rUTIs in premenopausal women independent of metabolic syndrome
***C. difficile***				
Papić et al. [60]	retrospective cohort study	314 patients ≥ 65 years, treated with antimicrobial therapy ≥ 24 h, and hospitalized ≥ 72 h in a 36-month period	- 16% of the patients with NAFLD and 7.4% of patients in the control group developed in-hospital CAD	- NAFLD is an independent predictor of CAD
Nseir et al. [61]	retrospective	115 patients with CAD vs. 115 patients without	- NAFLD was found in 66% patients with CAD vs. 30.4% in the control group - analysis showed that NAFLD was significantly associated with CAD	- NAFLD is a risk factor for CAD
Šamadan et al. [62]	retrospective cohort study	329 patients ≥ 60 years hospitalized with CAD, outcome: rCAD within 3 months of hospital discharge	- age > 75 years, NAFLD, CACI > 6, chronic kidney disease, statins and immobility were associated with rCAD	- NAFLD is a possible host-related risk factor associated with recurrent CAD
**Bacteremia and recurrent bacterial infections**				
Nseir et al. [63]	retrospective	247 patients with NAFLD hospitalized with bacterial infection vs. 100 patients without NAFLD	- NAFLD patients had significantly more RBIs-NAFLD, serum 25(OH)D level <20 ng/mL, obesity, were associated with RBIs, irrespective of MetS	- regardless of MetS, NAFLD is linked to an elevated risk of RBIs - NAFLD is frequently accompanied by vitamin D deficiency, which is also linked to an elevated risk of RBIs
Nseir et al. [66]	retrospective	71 patients with PB and hepatic ultrasonography were included	- the majority of patients with PB were found to show indications of NAFLD	- patients with PB presumed of gastrointestinal origin were associated with NAFLD
Gjurašin et al. [67]	retrospective cohort study	102 patients with invasive GBS	- in-hospital mortality was higher in patients with NAFLD	- NAFLD is associated with higher mortality in patients suffering from invasive GBS disease
**Hepatitis B, C, HIV**				
Adinolfi et al. [68]	review paper		- HCV-associated NAFLD has accelerated development of NASH, hepatic fibrosis, and hepatocellular carcinoma (HCC)	- some extrahepatic symptoms of chronic HCV infection are influenced by HCV-associated steatosis
Maurice et al. [69]	prospective study	patients with NAFLD and HIV monoinfection matched to HIV-positive and HIV-negative controls.	- cases with NAFLD were more obese and had significantly increased levels of sCD14, sCD163 and higher leptin to adiponectin ratio vs. Controls - cases with ≥F2 vs. < F2 fibrosis had increased sCD14 and sCD163 which correlated with waist circumference	- NAFLD fibrosis stage in HIV monoinfected patients is associated with monocyte activation in the context of obesity - may be independent of bacterial translocation and gut microbiome
Joo et al. [70]	cohort study	83,339 participants without NAFLD underwent serologic testing HBsAg between 2002 and 2006 and were followed annually or biannually until December 2014.	- during 484,736.1 person-years of follow-up, 20,200 incident NAFLD cases were identified - the adjusted hazard ratio for incident NAFLD comparing HBsAg-positive to HBsAg-negative participants was 0.83	- HBsAg seropositivity was associated with lower risk of developing NAFLD - possible effect of HBV infection on the pathogenesis of NAFLD development
**Periodontitis**				
Yoneda et al. [72]	retrospective observational study	the detection frequencies of periodontal bacteria in oral samples collected from 150 biopsy proven NAFLD patients and 60 non-NAFLD control subjects were determined	- the detection frequency of *P. gingivalis* in NAFLD patients was significantly higher than that in the non-NAFLD control subjects	- infection with high-virulence *P. gingivalis* might be an additional risk factor for the development/progression of NAFLD/NASH.

## Data Availability

All data are publicly available.
